# Intrascrotal Testicular and Extratesticular Epidermoid Cysts: About Two Cases

**DOI:** 10.5334/jbsr.2951

**Published:** 2022-11-10

**Authors:** Jean-Sebastien Bertrand, Ana Falticeanu, Olivier Lebecque

**Affiliations:** 1 Université catholique de Louvain, CHU UCL Namur, Department of Radiology, 1 Avenue Dr G Thérasse, 5530, Yvoir, BE

**Keywords:** epidermoid cyst, scrotum, testis

## Abstract

Painless solid testicular masses on ultrasonography are commonly malignant. However, if the lesion is well demarcated, rounded, and hypoechoic with alternating hyperechoic and hypoechoic layers, and no internal vascular flow, the possibility of an epidermoid cyst should be considered. Epidermoid cysts are uncommon benign testicular lesions and are extremely rare in the intrascrotal extratesticular region. Including these cysts in the differential diagnosis may allow the urologist to perform testis-sparing surgery.

**Teaching Point:** The possibility of an epidermoid cyst should be considered when a scrotal mass shows an ‘onion ring’ appearance on sonography and no vascularity on Doppler.

## Introduction

Epidermoid cysts (ECs) are uncommon benign tumors. On ultrasonography, especially in young men, finding a testicular mass should raise suspicion for malignancy. However, in view of certain sonographic characteristics, that is, an ‘onion ring’ appearance and lack of vascularity on color Doppler imaging, ECs should be included in the differential diagnosis.

## Case Histories

### Case 1

**Figure 1:**
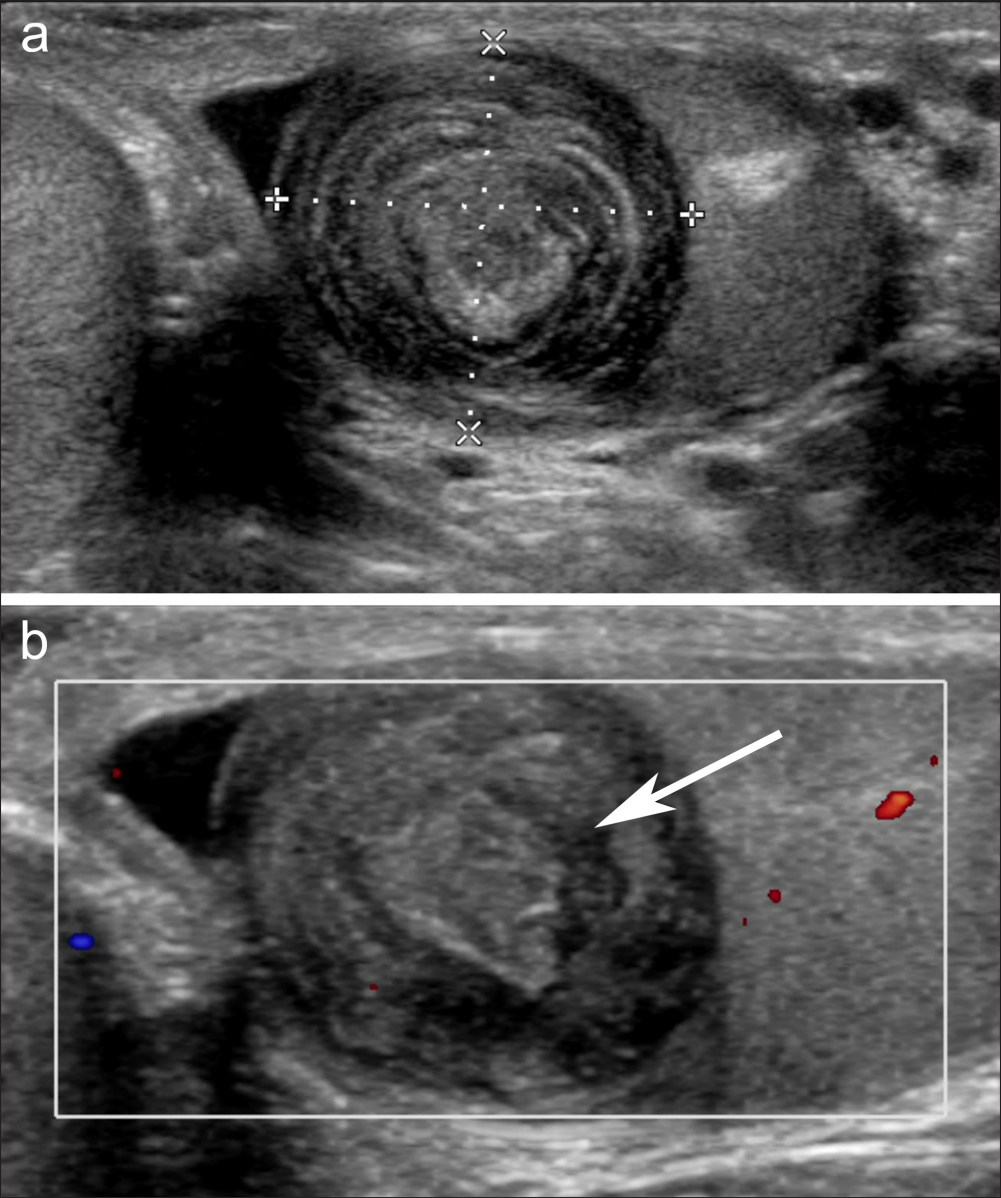
**(a)** B-mode sonography of the left testis shows an intratesticular mass with concentric rings of alternating echogenicity with a more echogenic central core. **(b)** Color Doppler sonography shows the absence of a vascular signal within the mass and a more echogenic central core (arrowhead).

A 25-year-old male was referred for sonographic evaluation after noticing a painless swelling in the left testis. Ultrasonography showed a hypoechoic, well-circumscribed, rounded 17 mm mass in the left testicle. The mass showed alternating rings of hypo- and hyperechogenicity with an echogenic central core (Figure 1a). Color Doppler imaging showed no internal vascular flow (Figure 1b). The diagnosis of an epidermoid cyst was suggested. Laboratory tests for alpha-fetoprotein (AFP) and beta-human chorionic gonadotrophin (HCG) were negative. Surgery was performed and pathology confirmed the diagnosis of a testicular EC.

### Case 2

**Figure 2:**
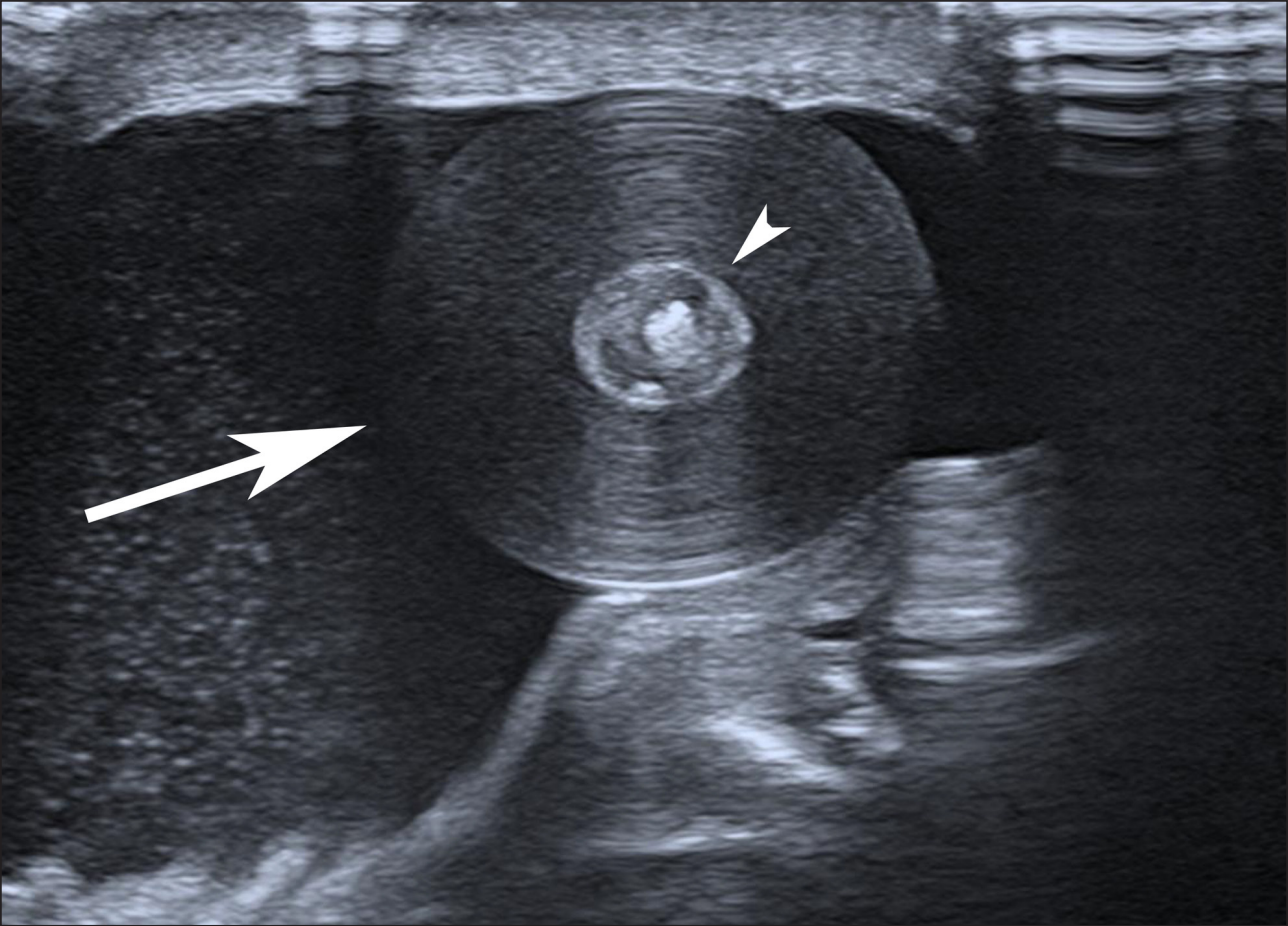
Ultrasonography of the right hemiscrotum shows a mild hydrocele and an extratesticular intrascrotal mass with concentric rings of alternating echogenicity. Note the hyperechogenic central core (arrowhead).

A 58-year-old male consulted for a right-sided painless scrotal nodule present for years. The test for the HCG marker was negative, but his AFP was elevated (60.4 µg/L; normal <7.51). Ultrasonography revealed a right well-circumscribed, rounded intrascrotal extratesticular mass measuring 17 mm, associated with a moderate hydrocele. The lesion demonstrated an ‘onion ring’ appearance with an echogenic central target appearance and was avascular on color Doppler sonography (Figure 2, Video 1). Both testes were normal. The diagnosis of an epidermoid intrascrotal extratesticular epidermoid cyst was suggested. Further investigation indicated recurrence of hepatocarcinoma as an explanation for the elevated AFP. The urologist recommended watchful waiting for as long as the lesion remained painless.

**Video 1:** Ultrasound video of a right hemiscrotum extratesticular lesion showing characteristic “onion ring” appearance with a mild hydrocele.

## Discussion

ECs are keratin-filled cysts, accounting for between 1 and 2% of all testicular tumors [1,2]. The most common presenting symptom is a palpable painless nodule [2]. ECs may manifest clinically as mild pain or discomfort. However, they are generally asymptomatic and often go undetected until discovery during a routine physical examination [1]. In a recent study, the median age of patients with ECs was 26 years, including prepubertal patients (range 11–34 years). The reported mean diameter of ECs was 1.74 cm [2]. ECs can appear as multiple and/or bilateral cysts and are very rarely extratesticular [3,4].

The sonographic findings include well-demarcated round-shaped lesions with an ‘onion ring’ pattern consisting of alternating hyperechoic and hypoechoic layers [5,6,7]. However, this feature is not always observed, with Manning et al. reporting it in 13 of 21 lesions (62%) [5]. In this series, the most commonly reported characteristics were mural calcifications (86%) ranging from scattered foci to dense calcifications. The third most common pattern (33%) included a hypoechoic rim with an echogenic central core—a target appearance. Color-Doppler imaging should be performed and does not show central flow at appropriate sensitive settings. Despite their solid appearance, ECs are considered to be cysts. Features such as poorly defined borders and internal heterogeneity should raise suspicion of a malignancy [5].

While ECs are benign lesions, they can be mimicked by epidermoid cysts developing in association with invasive germ cell tumors, thus representing a specific differentiation in teratomas, analogous to the occurence of other intratumoral benign structures, such as hairs or teeth. Anheuser et al. reported two such cases with preoperative imaging detecting only ‘simple’ ECs that were later associated with embryonal carcinoma in one patient and a germ cell neoplasia in situ in the other patient [2].

A suspicion of EC by the radiologist may allow the urologist to proceed with a frozen section examination, biopsies of the surrounding parenchyma, and possibly testis-sparing surgery instead of an intentionally planned orchiectomy [2,5].

## Conclusions

On ultrasonography, a solid and painless solid intratesticular mass should raise suspicion of malignancy, especially in a young man. However, if the tumor markers are negative and if the lesion has a typical ‘onion ring’ sonographic pattern, well circumscribed and without vascularity on Color Doppler, ECs should be included in the differential diagnosis. If the lesion is confirmed to be an EC, the urologist may be able to perform testis-sparing surgery with frozen-section analysis rather than orchiectomy. Very rarely, ECs can be extratesticular in the cavity of the tunica vaginalis.
